# Translation and cultural adaptation of the I-CAM-Q: the first Hungarian version for assessing complementary and alternative medicine use

**DOI:** 10.1186/s12906-025-05220-2

**Published:** 2025-12-20

**Authors:** Tibor Rák, Anita Hegedűs, Eszter Molnár, Adrienne Csutak

**Affiliations:** 1 Department of Ophthalmology, Medical School, Clinical Centre, University of Pécs, Pécs, H-7624 Hungary; 2 Department of Languages for Biomedical Purposes and Communication, Medical School, University of Pécs, H-7624 Pécs, Hungary

**Keywords:** I-CAM-Q, Complementary and alternative medicine, Cross-sectional study, Linguistic and intercultural adaptation, Hungary, Questionnaire validation

## Abstract

**Background:**

The International Questionnaire to Measure the Use of Complementary and Alternative Medicine (I-CAM-Q) is a standardized tool for assessing CAM use. While it has been adapted into several languages, this study presents the first Hungarian translation and cultural adaptation. Given the growing interest in CAM in Hungary, a validated Hungarian version is essential for accurate data collection and informed healthcare policy.

**Methods:**

The I-CAM-Q was translated into Hungarian using a rigorous forward–backward translation protocol involving expert translators, proofreaders, and a reconciliation panel. The final version was pilot-tested among healthy volunteer healthcare workers from ophthalmology departments. The questionnaires were used to assess clarity, cultural relevance, and reliability in this cross-sectional study. Individuals with diagnosed eye diseases were excluded.

**Results:**

Among ophthalmologically healthy volunteers, 77.8% consulted physicians in the past 12 months, while 8.9% visited chiropractors and spiritual healers, and 2.2% consulted acupuncturists. In the past 3 months, physicians remained the most consulted (73.7%), followed by chiropractors (53.3%). CAM therapies were mainly used for general well-being and acute conditions, with high satisfaction 75–100% of users rated chiropractic, acupuncture, and spiritual healing as “very useful.” Phytomedicines were used primarily for acute illness (41.7%) and general well-being (27.1%), with 68.1% of users rating them as very useful. Dietary supplements were widely consumed, especially Vitamin D (62.9%) and Vitamin C (57.1%), mainly for prevention (46.2%) and general health (38.5%). Meditation and relaxation techniques were also commonly practiced, with 92.3% and 69.2% of users, respectively, rating them as very useful.

**Conclusions:**

The Hungarian version of the I-CAM-Q was successfully translated, culturally adapted, and validated. It provides a reliable tool for assessing CAM use in Hungary and supports further research into CAM practices and their integration into healthcare.

**Supplementary Information:**

The online version contains supplementary material available at 10.1186/s12906-025-05220-2.

## Introduction

Complementary and alternative medicine (CAM) encompasses a wide range of health practices not typically part of conventional medicine. The I-CAM-Q (International Complementary and Alternative Medicine Questionnaire) was developed by Sara Quandt et al. [1] to provide a comprehensive and comparable measurement of the use of CAM. The questionnaire aims to provide internationally standardized data for health system planning and understanding CAM usage. It is unique in that it evaluates CAM treatments recommended by physicians, self-administered practices, as well as the motivations and experiences associated with these treatments. The questionnaire was first validated in Germany and Switzerland, and subsequently widely used in other European countries such as France, Poland, Norway, Sweden, the Netherlands, Romania, and Spain [1–8]. It has been validated on almost every continent, including Asia (e.g., Iran, Saudi Arabia, Taiwan, Japan, and South Korea) [9–14]. The questionnaire has also been tested in Australia, as well as in the United States and Argentina on the American continent [2, 6, 15].

According to estimates by the World Health Organization (WHO), 60–80% of the world’s population, particularly in developing countries, relies on complementary medical practices to alleviate symptoms or manage their health conditions [16, 17]. While a significant portion of this use involves herbs or plant-based products, with 85% of individuals in these regions using herbal preparations [16, 17], CAM encompasses a much broader spectrum of practices. These include manual body-based therapies (e.g., chiropractic methods, massage, and naprapathy), mind-body interventions (e.g., meditation, yoga, and relaxation techniques), and complete traditional medical systems [e.g., Traditional Chinese Medicine (TCM) or Ayurveda], alongside the use of herbal, mineral, or animal-derived substances [8, 12, 13, 16]. In developed countries, there is also a growing trend of returning to classical ethnomedicinal practices, particularly herbal-based preparations, but also a significant uptake of other CAM forms. For instance, studies using the I-CAM-Q have shown high prevalence rates for manual therapy in Sweden and mind-body practices in Japan [8, 12]. Complementary medical practices globally often taught in medical and pharmaceutical education. The regular use of CAM therapies in European countries is as follows: 49% in France, 46% in Germany, 35% in the United Kingdom, 31% in Belgium, and 25% in Northern European countries [16, 18]. In most countries, these naturopathic methods are not officially recognized, but there is increasing demand for them from both patients and healthcare professionals.

While the I-CAM-Q has been adapted into several languages, this study represents the first translation and cultural adaptation of the questionnaire into Hungarian. The primary objective was to ensure the Hungarian version is both linguistically and culturally appropriate, and to validate its use among healthy volunteers. The importance of this adaptation lies in the growing interest in and use of CAM in Hungary. With an increasing number of individuals turning to CAM for various health issues, it is crucial to have a reliable tool to evaluate its usage and effectiveness. The Hungarian adaptation of the I-CAM-Q will enable researchers and healthcare providers to gather accurate data on CAM practices, which can inform healthcare policies and improve patient care.

## Methods

### Questionnaire translation

**Fig. 1:**
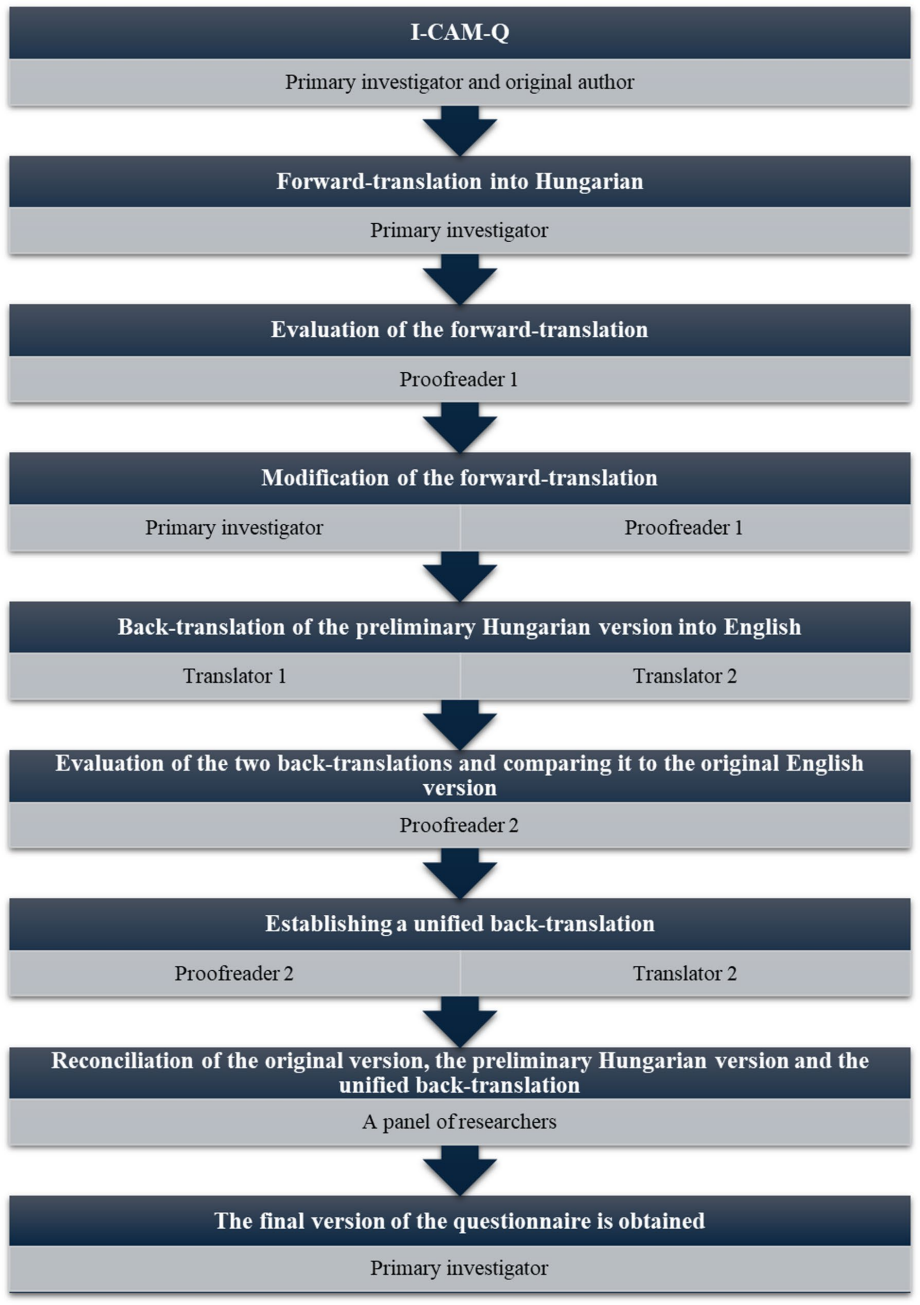
Methods of translation and linguistic validation and the cross-cultural adaptation process of the Hungarian version of the I-CAM-Q questionnaire

The primary investigator contacted the main author of the original version of Norway’s National Research Centre for Complementary and Alternative Medicine (*NAFKAM*) *International CAM Questionnaire (I-CAM-Q)* and obtained permission for translating and using the questionnaire in the study (both the original English I-CAM-Q and the validated Hungarian translation are available in the Appendix). The questionnaire was submitted for validation. Prior to the research, the questionnaire was adapted into Hungarian with the assistance of the Department of Languages for Biomedical Purposes and Communication of the Faculty of Medicine at the University of Pécs, following the standard translation and cultural adaptation protocol. This protocol includes translation, back-translation, review by an expert panel, and pilot testing in a cohort of healthy volunteers from various age groups. According to the Department of Languages for Biomedical Purposes and Communication, University of Pécs, Medical School the process of questionnaire validation is the following (Fig. 1): During the validation process, this protocol was strictly observed. As a first step, the primary investigator translated the original questionnaire from English into Hungarian. The primary investigator is a medical specialist and a native speaker of the target language (Hungarian) with an advanced-level command of English. The translation was reviewed by Proofreader 1, who is a native speaker of the target language (Hungarian) and a professional medical English teacher and translator with a PhD in linguistics. Changes were made after evaluation and discussion and a preliminary Hungarian version was agreed on by the primary investigator and Proofreader (1) Subsequently, the preliminary Hungarian version was back-translated into English by two experienced translators who are both professional medical English teachers and translators, one of them with a PhD in linguistics. Both translations were evaluated and compared to the original questionnaire by Proofreader (2) Proofreader 2 is a native speaker of English, who speaks Hungarian as a second language and is a professional medical English teacher. Following evaluation by Proofreader 2, the two translators and Proofreader 2 agreed on a unified back-translated version. Eventually, an online reconciliation of the original version, the back translations and the preliminary Hungarian translation was conducted by a panel involving the primary investigator, the two translators and the two proofreaders. Finally, as a result of the reconciliation, a consensus harmonized Hungarian final version of the questionnaire was obtained.

### Participant selection and rationale

The target group for this initial validation research consists of healthy volunteer healthcare workers (*n* = 49) from the University of Pécs, Clinical Centre, Department of Ophthalmology and Mohács Hospital Outpatient Department of Ophthalmology. The questionnaire was made available both online and in print to obtain a representative sample. General demographic and epidemiological data were collected to assess statistical accuracy, based on the methodology of Yurdakul and Al Salman et al. [19, 20], supplemented and modified with content relevant to our study, which does not include identifiable information of anonymous respondents. At the end of the survey, we included a “*Comments and Feedback*” section to capture the general impressions of the respondents regarding the questionnaire, the healthcare facility and ophthalmic care. Exclusion criteria included pre-existing eye diseases. Spectacle correction in the case of best corrected visual acuity 20/20 in each eye is not considered as eye disease.

This sampling strategy was implemented for two primary reasons: first, from a feasibility and methodological standpoint, recruiting a cohort without diagnosed ocular pathology allowed for the efficient establishment of a baseline for the questionnaire’s performance. Access to this participant pool was readily available through our institutional network, facilitating timely data collection for this pilot study. Furthermore, the absence of active disease minimizes the potential confounding effects of medication, recent surgical interventions, or condition-specific health-seeking behaviors on the general patterns of CAM use, thereby providing a clearer initial validation of the instrument itself. Second, and most critically, this approach aligns with the future application of the adapted instrument. As the ultimate objective is to deploy this questionnaire in ophthalmic patient populations, its content was supplemented with ophthalmology-specific items. Validating the tool in a healthy population represents a necessary first step in establishing its fundamental psychometric properties (such as internal consistency and factor structure) before its application in more complex, clinically affected cohorts. This sequential validation process ensures that the instrument is robust and reliable prior to investigating the relationships between CAM use and specific eye conditions.

### Data collection and analysis

To assess the reliability of the Hungarian translation of the I-CAM-Q, we employed mixed-methods approach, combining quantitative and qualitative analysis. Upon completion of the survey, participants were provided with an optional, free-text (“*Comments and Feedback*”) to share their general impressions, evaluating the clarity, cultural relevance, and interpretability of the translated items. A semi-structured interview guide was used to explore the following domains: the perceived relevance and familiarity of various CAM therapies listed in the questionnaire within the Hungarian context were discussed. Feedback on the length, structure, and flow of the entire questionnaire was gathered. The qualitative feedback from these interviews was analyzed thematically. The primary finding was the perceived length of the questionnaire, which was noted as a minor burden. However, no major misunderstandings or systematic issues with item comprehension were identified, confirming the conceptual and linguistic adequacy of the translated version. As a result, no further revisions to the questionnaire items were deemed necessary.

Data analysis was conducted using Microsoft^®^ Excel^®^ for Microsoft 365, version 16.0.19029.20136 (32-bit), developed by Microsoft Corporation (Redmond, WA, USA). The sample size (*n*) for each therapy presented in the tables and the text reflects the number of valid responses for that specific item. As participants could skip questions or using multiple certain therapies at the same time, these sample sizes vary. All statistical analyses were conducted using these item-specific response counts. To assess associations between categorical variables (gender, age groups) and the use of specific CAM therapies, Fisher’s exact tests were employed. This non-parametric test was chosen due to its appropriateness for small sample sizes and its accuracy when expected cell counts in 2 × 2 contingency tables are below 5. A statistical significance level was defined as *p* < 0.05. The analysis focused on examining potential differences in therapy utilization patterns between males and females, as well as between participants below and above 35 years of age.

## Results

### Sociodemography and preferences for complementary medicine in ophthalmology

The following section presents the key findings of the Hungarian adaptation of the I-CAM-Q questionnaire, including the sociodemographic profile in Table 1. The final sample consisted of 49 participants. The study population consisted of 29 females (59%) and 20 males (41%). A significant association was found between gender and age distribution (Fisher’s exact test, *p* = 0.045). While males constituted 41% of the total sample, they represented a significantly higher proportion (64%, 7/11) of the under-35 age group compared to the older cohort (34%, 13/38). In contrast, no significant association was observed between gender and the highest level of educational attainment (*p* = 1.0). The gender distribution was nearly identical among participants with secondary education (41% male, 59% female) and those with a higher education degree (41% male, 59% female). According to the self-assessed health status data, 21% of respondents rated their health as excellent, 19% as very good, 42% as good, 16% as fair, and 2% (1 person) as poor (Table 1). The results indicate that the majority of respondents are in good health, with a combined 40% rate as excellent or very good. The proportion of those reporting fair or poor health is relatively low. Despite most informants being in good or excellent health, a significant proportion still have at least one known illness: the data shows that more than half of the respondents, specifically 58%, have at least one known general disease, while 42% reported no such condition. This suggests that the presence of known illnesses does not necessarily have a substantial impact on individuals’ overall self-assessed health status. High blood pressure is the most prevalent condition, affecting 34% of the respondents. Diabetes follows, with 19% of the respondents reporting this condition. Asthma is less common, with only 6% of respondents affected. Cancerous conditions are reported by 13% of the respondents. Additionally, 34% of the respondents indicated having other types of illnesses not specified in the main categories. This distribution highlights the significant burden of high blood pressure and diabetes within the surveyed population. The relatively high percentage of respondents with other unspecified illnesses (e.g. hypothyroidism, migraine, rheumatoid arthritis, depression, cholelithiasis etc.) suggests a diverse range of health issues that may require further investigation.

**Table 1:** Summary of sociodemographic factors among ophthalmologically healthy Hungarian participants of the I-CAM-Q questionnaire

Variable	*Number of informants* (*n* = 49; %)
Gender	
Male	20 (41%)
Female	29 (59%)
*Age (years)*	
18–24 years	2 (4%)
25–34 years	9 (18%)
35–44 years	15 (31%)
45–54 years	10 (20%)
55–64 years	9 (18%)
Above 65 years	4 (8%)
*Highest qualification*	
Primary school	0 (0%)
Secondary school	22 (45%)
University/college degree	25 (51%)
Postgraduate education	2 (4%)
*Monthly income*	
$0 - $1,400 USD	25 (51%)
$1,400 - $2,800 USD	19 (39%)
$2,800 USD	5 (10%)
*Officially registered residence (Hungarian county or Budapest)*	
Budapest	7 (14%)
Baranya	34 (69%)
Békés	1 (2%)
Hajdú-Bihar	1 (2%)
Pest	1 (2%)
Somogy	1 (2%)
Vas	3 (6%)
Zala	1 (2%)
*Self-assesed health status*	
Excellent	11 (21%)
Very good	10 (19%)
Good	21 (42%)
Fair	8 (16%)
Poor	1 (2%)

Among the voluntary respondents, individuals with significant visual impairment or those requiring ophthalmic treatment were excluded beforehand. Consequently, 73% (*n* = 41) of the respondents self-reported having no eye disease. The other 27% (*n* = 8) considered their correctable presbyopia or myopia eye disease, however, refractive errors that can be corrected to 20/20 best corrected visual acuity (BCVA) with spectacles are not considered diseases from a professional standpoint.

The survey included a question regarding the prevalence of diagnosed eye diseases among respondents’ relatives, allowing for multiple responses. This aims to determine whether the respondents are aware of any eye diseases in their genetic ancestors. The data reveal that the most common conditions are those correctable with spectacles, reported by 19 respondents (31%). Cataract follows, affecting the family members of 15 respondents (25%), while an equal proportion (25%) reported no known eye disease in their families. Seven individuals (11%) indicated a family history of glaucoma, and two (3%) reported relatives suffering from dry eye disease. Known age-related macular degeneration (AMD) and “other” conditions were each reported by one respondent (2%), while no cases of diabetic retinopathy were reported. These findings highlight the significant presence of correctable conditions and common eye diseases among the surveyed population and their families.

The inquiry into respondents’ familiarity with alternative treatments for eye diseases indicates that 57% (*n* = 28) are aware of such treatments, whereas 43% (*n* = 21) are not. This suggests a considerable level of awareness regarding alternative therapies within the surveyed group. This considerable awareness can likely to be attributed to the pervasive influence of the modern wellness movement and direct-to-consumer marketing. Widespread information on supplements like bilberry and lutein for eye health, coupled with a growing public interest in proactive self-care and complementary approaches, has effectively disseminated this knowledge beyond specialized circles, making it familiar to a majority of the general public.

The following investigates the sources from which respondents gather information about complementary and alternative therapies for eye diseases. The findings indicate that healthcare professionals are the primary source, with approximately 30% of respondents (*n* = 20) citing them. Social media platforms, such as Facebook, are the second most common source, mentioned by about 23% of respondents (*n* = 13). Additionally, family, friends, and acquaintances serve as significant sources, with around 15% of respondents (*n* = 10) relying on them. In contrast, 23% of respondents (*n* = 15) do not seek information about these complementary therapies. These findings suggest that while there is a considerable awareness of alternative treatments for eye diseases, the sources of information vary widely. Healthcare professionals play a crucial role in disseminating information, but social media and personal networks also contribute significantly. This highlights the importance of ensuring that accurate and reliable information about alternative therapies is available across multiple channels to support informed decision-making among individuals.

The data on the respondents’ preferences regarding the acceptance of complementary and alternative therapies from various professionals reveals that a significant majority, 56% of respondents (45 individuals), would accept such therapies from a medical doctor. This indicates a strong preference for receiving complementary treatments from professionals with formal medical qualifications. This group likely values conventional medical training and may be skeptical of alternative treatments. Additionally, 25% of respondents (*n* = 20) are open to accepting complementary therapies from non-medical professionals. This suggests a notable level of trust in alternative practitioners who may not have a medical degree but are still considered knowledgeable in their field. Interestingly, 14% of respondents (*n* = 11) would accept complementary therapies from any therapist without a specific healthcare qualification. These respondents believe that experience and knowledge in certain complementary therapeutic procedures are not strictly tied to a healthcare qualification, as long as there is trust in the knowledge and the therapist-patient relationship. Lastly, 5% of respondents (*n* = 4) would not accept complementary therapy regardless of the therapist’s qualifications. These findings highlight the importance of medical qualifications in the acceptance of complementary and alternative therapies. The data suggests that while there is an openness to alternative treatments, there is a clear preference for these therapies to be administered by medically trained professionals. This underscores the need for integrating complementary therapies within the framework of conventional medical practice to enhance their credibility and acceptance among the general population.

The investigation into whether respondents would discuss their use of complementary and alternative treatments with their ophthalmologist reveals that only 21% (*n* = 3) are inclined to do so. Conversely, a significant majority of 71% (*n* = 10) would not discuss these treatments with any ophthalmologist, while 7% (*n* = 1) prefer to consult another doctor instead. This indicates that while some patients are open to discussing complementary therapies, many prefer to seek advice from healthcare providers other than their ophthalmologists. Rooted in the traditional, authority-oriented culture of Hungarian healthcare, patients’ deep respect for physicians, particularly specialists like ophthalmologists, often discourages them from discussing CAM practices. This respect, combined with an expectation of medical skepticism towards CAM, often leads patients to fear dismissal. The resulting ‘closet effect’ conceals a significant part of their health behavior, creating a critical communication gap. For instance, a patient might never mention using an herbal supplement for fear of their ophthalmologist’s judgment, potentially hindering comprehensive care.

Subsequently, we aimed to investigate the reasons why respondents chose not to discuss their complementary treatments with their ophthalmologist. The primary reason, cited by 60% (6 respondents), is the concern that the ophthalmologist lacks competence in complementary treatments. Additionally, 40% (*n* = 4) provided other reasons, such as not needing complementary therapies or not having eye diseases. These findings suggest that a significant barrier to discussing complementary treatments with ophthalmologists is the perception of the doctor’s insufficient understanding or expertise in this area. This underscores the need for improved communication and education regarding complementary therapies within the medical community, ensuring patients feel comfortable discussing all aspects of their treatment with their healthcare providers.

In examining treatment preferences when an ophthalmologist offers both evidence-based medicine and complementary/alternative treatments for an eye disease, the responses reveal varied inclinations. Specifically, 20% of respondents (*n* = 10) would favor evidence-based treatments, while 8% (*n* = 4) would choose complementary/alternative treatments. A significant majority, 49% (*n* = 24), would accept both types of treatments, indicating a willingness to integrate conventional and alternative approaches. Additionally, 22% of respondents (*n* = 11) would defer the decision to the medical professional, relying on their expertise and experience. These findings underscore a general openness to combining traditional and alternative therapies, reflecting a nuanced approach to healthcare where patients are willing to explore multiple avenues for treatment.

The study examines the financial commitment respondents are willing to make towards complementary and alternative treatments. The data reveals that 59% of respondents (*n* = 29) would allocate less than 10% of their monthly salary to such treatments. Conversely, 41% of respondents (*n* = 20) are open to higher monthly expenditures, provided the treatments are effective. This indicates that while there is substantial interest in complementary and alternative therapies, cost remains a significant consideration for most individuals. However, a notable segment of the population is willing to invest more in these treatments if they perceive them to be effective, suggesting a potential market for high-quality alternative therapies that demonstrate clear benefits.

### I-CAM-Q

#### Healthcare services

The initial domain assessed by the I-CAM-Q questionnaire pertains to the frequency of consultations with various healthcare providers over the preceding 12 months, encompassing both conventional medical practitioners and those offering complementary and alternative therapies.

This study collected and analyzed data on the types and frequencies of healthcare providers visited by participants (Table 2), enabling comparison of healthcare-seeking patterns. In the past 12 months (*n* = 45), the most commonly consulted healthcare provider was the physician (77.8%), followed by chiropractors (8.9%), spiritual healers (8.9%), and acupuncturists (2.2%). Homeopaths, physiotherapists, and visual trainers were not consulted at all during this period. One participant (2.2%) visited a pharmacist (categorized as “Other”) two times. A notable gender disparity was observed in the utilization patterns of specific CAM providers. While male participants visited physicians, chiropractors, and pharmacists, they did not consult other practitioners, such as acupuncturists or spiritual healers. This suggests that men’s engagement with complementary medicine may be limited to providers operating within a more conventional, biomechanical paradigm. The complete absence of men seeking services perceived as more esoteric or holistic may reflect deeply ingrained gender norms in health-seeking behavior and the perceived legitimacy of different therapeutic approaches.

**Table 2:** Summary of visits to healthcare providers among the survey informants

Visited healthcare providers in the past 12 months		
	*n* = 45	Percentage (%)
Physician	35	77.8
Chiropractor	4	8.9
Homeopath	0	0.0
Acupuncturist	1	2.2
Phytotherapist	0	0.0
Spiritual healer	4	8.9
Visual trainer	0	0.0
Other (Pharmacist)	1	2.2

Visited healthcare providers in the past 12 months		
	*n* = 57	Percentage (%)
Physician	42	73.7
Chiropractor	6	10.5
Spiritual healer	4	7.0
Pharmacist	2	3.5
Acupuncturist	2	3.5

A distinct pattern emerged among participants under 35 years of age: while they visited physicians, they did not utilize any other CAM therapists, such as chiropractors or acupuncturists. This suggests that younger individuals in this cohort may primarily rely on the conventional healthcare system and have not yet integrated specialist complementary providers into their health management strategies. This could be attributed to several factors. Younger adults may have less exposure to or awareness of the specific benefits of different CAM modalities. Furthermore, they might not yet experience the chronic health issues that often lead individuals to explore alternatives beyond standard medical care.

In the preceding 3 months (*n* = 57), physicians also remained the most frequently visited providers (73.7%). Chiropractors were consulted by 10.5% of participants, spiritual healers by 7.0%, pharmacists by 3.5%, and acupuncturists by 3.5%. These findings indicate a predominant reliance on conventional medical care (physicians), with a notable but smaller proportion of the population also utilizing complementary or alternative health services, particularly chiropractic and spiritual healing among ophthalmologically healthy population.

No significant associations were found between the utilization of physicians or chiropractors within the 12 months and either gender or age when analyzed using Fisher’s exact test. Specifically, physician visits showed no significant association with gender (*p* = 0.714) or age group (*p* = 1.0). Similarly, chiropractor visits were not significantly associated with gender (*p* = 0.607) or age group (*p* = 0.556). The frequent utilization of these providers was independent of both age and gender.

Among the 35 individuals who consulted physicians, the most frequent reasons were acute diseases (42.9%) and chronic conditions (34.3%), followed by occupational health check-ups (17.1%) and general well-being (5.7%) (Table 3). Chiropractors (*n* = 4) were primarily visited for general well-being (50%), acute disease (25%), and chronic disease (25%) (Table 3). The single acupuncturist consultation was related to an acute disease (100%) (Table 3). Spiritual healers (*n* = 4) were consulted mainly for general well-being (50%), and equally for acute (25%) and chronic conditions (25%) (Table 3). One participant consulted another provider (pharmacist) for an acute illness (100%). Homeopaths, phytotherapists, and visual trainers were not consulted by any participants in this dataset (Table 3). Physician services were rated as “very useful” by 45.7% and “moderately useful” by 31.4% of users, with 11.4% rating them as “not useful at all” (Table 3). Chiropractor services were rated as “very useful” by 75% of respondents and “moderately useful” by 25% (Table 3). All users of acupuncture and spiritual healing services (100%) found them to be “very useful.” The sole user of pharmacist services also rated them as “very useful” (Table 3). These findings suggest that while physicians are consulted for a wide range of medical needs, complementary providers like chiropractors and spiritual healers are often sought for well-being and are perceived as beneficial by their users. The strikingly highly perceived usefulness of these CAM modalities, despite their limited use for severe disease, suggests that participants value them for different, potentially non-medical outcomes such as stress reduction, personal empowerment, or holistic balance. This underscores that the choice of a healthcare provider is not merely a clinical decision but is deeply influenced by the individual’s goals, whether they are disease-specific treatment or the enhancement of overall wellness.

**Table 3:** Distribution of primary reasons for consulting different healthcare providers and subjective evaluation of the perceived benefits of these services. Percentages (%) reflect the proportion of respondents reporting each reason or level of usefulness

	Physician		Chiropractor		Homeopath		Acupuncturist		Phytotherapist		Spiritual healer		Visual trainer		Other	
	*n* = 35	%	*n* = 4	%	*n* = 0	%	*n* = 1	%	*n* = 0	%	*n* = 4	%	*n* = 0	%	*n* = 1	%
*Primary reason for consulting healthcare providers*																
General well-being	2	5.7	2	50.0	0	0.0	0	0.0	0	0.0	2	50.0	0	0.0	0	0.0
Acute disease	15	42.9	1	25.0	0	0.0	1	100.0	0	0.0	1	25.0	0	0.0	1	100.0
Chronic disease	12	34.3	1	25.0	0	0.0	0	0.0	0	0.0	1	25.0	0	0.0	0	0.0
Other (occupational health check-up)	6	17.1	0	0.0	0	0.0	0	0.0	0	0.0	0	0.0	0	0.0	0	0.0
*Subjective evaluation of perceived benefit from healthcare services*																
Very useful	16	45.7	3	75.0	0	0.0	1	100.0	0	0.0	4	100.0	0	0.0	1	100.0
Moderately useful	11	31.4	1	25.0	0	0.0	0	0.0	0	0.0	0	0.0	0	0.0	0	0.0
I do not know	1	2.9	0	0.0	0	0.0	0	0.0	0	0.0	0	0.0	0	0.0	0	0.0
Not useful at all	4	11.4	0	0.0	0	0.0	0	0.0	0	0.0	0	0.0	0	0.0	0	0.0

Twelve respondents (*n* = 12) reported participating in therapy sessions from a naturopathic doctor. The most commonly provided treatment was body manipulations (manual therapy), reported by 58.3% (*n* = 7) of informants. Phytotherapy was the second most frequent treatment, used by 16.7% (*n* = 2) of naturopathic doctors (Table 4). Acupuncture, spiritual healing, and dietary modification were reported by 8.3% (*n* = 1) of respondents, respectively, while homeopathy and visual training were not practiced by any of the surveyed professionals. Regarding therapy sessions (*n* = 20) over the preceding three months, manual therapy accounted for the vast majority (95%; *n* = 19) of the sessions, indicating its dominant role in naturopathic care. Phytotherapy was applied in 5% (*n* = 1) of the cases (Table 4). These findings suggest that manual therapy is not only the most commonly offered treatment among naturopathic doctors but also the most frequently utilized one in practice. The overwhelming dominance of manual therapy reveals a significant practical narrowing of naturopathic practice within this sample. While naturopathy is philosophically rooted in a wide array of holistic and natural treatments, the actual care provided appears to converge heavily on a single, structurally-focused modality. This raises important questions about the de facto professional identity of these practitioners in the local context. Are they acting as broad-spectrum naturopaths or have they effectively specialized as manual therapists operating under a naturopathic title? This discrepancy between philosophical breadth and practical focus could reflect patient demand for tangible, physical interventions, the practitioners’ own specialized training, or market-driven adaptation to what is most recognized and valued by their clients.

**Table 4:** Therapeutic interventions administered by naturopathic doctors over the past year (*n* = 12) and distribution of therapy sessions over the last three months (*n* = 20)

Types of treatments provided by naturopathic doctors in the last 12 months		
	*n* = 12	%
Manual therapy	7	58.3
Homeopathy	0	0.0
Acupuncture	1	8.3
Phytotherapy	2	16.7
Spiritual healing	1	8.3
Visual training	0	0.0
Other (Dietary modification)	1	8.3

*Number of therapy sessions with naturopathic doctors over the last three months*		
	*n* = 20	%
Manual therapy	19	95.0
Phytotherapy	1	5.0

The study examined the utilization patterns of selected naturopathic therapies, along with the self-reported reasons for consultating naturopathic doctors over a defined observation period. Homeopathy and visual training were excluded from the analysis due to a lack of reported use among participants. The most frequently utilized therapy was manipulation (*n* = 7), followed by acupuncture (*n* = 2), phytotherapy (*n* = 1), spiritual healing (*n* = 1), and other therapies (*n* = 2). The primary reason for seeking naturopathic care was general well-being, particularly in the manipulation group, where 62.5% of respondents indicated this as their main motivation (Table 5). One respondent marked both chronic disease and general well-being as main indications. Dietary modification was indicated as the only answer in the category “Other”. Manual therapy emerged as the most frequently assessed intervention, with 75% of respondents (*n* = 6) rating it as “very useful” and an additional 25% (*n* = 2) as “moderately useful.” No negative or uncertain evaluations were reported, indicating a high level of perceived therapeutic benefit. Acupuncture demonstrated a comparable pattern, with 75% of participants rating it as “very useful” and 25% as “moderately useful,” suggesting similarly favorable perceptions. Phytotherapy and dietary modification were each rated as “very useful” by a single respondent, though no further evaluations were available. One participant rated spiritual healing as “not useful at all,” indicating divergent perceptions of complementary therapies (Table 5).

**Table 5:** Main motivations for seeking naturopathic care and perceived therapeutic outcomes as reported by patients. Homeopathy and visual training were omitted because they were not reported by participants

	Manual therapy		Acupuncture		Phytotherapy		Spiritual healing		Other^a^	
	*n* = 8	%	*n* = 1	%	*n* = 2	%	*n* = 1	%	*n* = 1	%
*Main reason for seeking consultation with a naturopathic doctor*										
General well-being	5	62.5	0	0.0	1	50.0	0	0.0	1	100.0
Acute disease	1	12.5	1	100.0	0	0.0	0	0.0	0	0.0
Chronic disease	2	25.0	0	0.0	1	50.0	1	100.0	0	0.0
*Self-reported evaluation of therapeutic benefit from naturopathic medical treatment*										
Very useful	6	75.0	1	100.0	1	50.0	0	0.0	1	100.0
Moderately useful	2	25.0	0	0.0	1	50.0	0	0.0	0	0.0
I do not know	0	0.0	0	0.0	0	0.0	0	0.0	0	0.0
Not useful at all	0	0.0	0	0.0	0	0.0	1	100.0	0	0.0

#### Supplements and medicines

Participants in the survey provided diverse responses regarding the herbal products consumed in the preceding 12 months (Table 6). A total of 22.4% of respondents (*n* = 11) reported consuming a specific commercially available herbal dietary supplement, while 6.1% (*n* = 3) opted for a particular brand of tea mixture. Additionally, 32.6% (*n* = 16) consumed monoherbal tea preparations either periodically or regularly, listing various herbs such as *Acorus calamus* L. (Acoraceae), *Ribes nigrum* L. (Grossulariaceae), *Plantago lanceolata* L. (Plantaginaceae), *Juglans regia* L. (Juglandaceae*)*, etc. Several herbs were mentioned by multiple participants, with the most popular being *Matricaria recutita* L. (Asteraceae) (8.16%) and *Withania somnifera* (L.) Dunal (Solanaceae) (6.1%) among ophthalmologically healthy volunteers. Among the herbal products reported by participants, 65.3% (*n* = 32) were indicated as currently in use at the time of the survey.

**Table 6:** Distribution, primary reason and subjective evaluation of herbal products consumed in the past 12 months by survey informants

Herbal products applied in the past 12 months		
	Products *(n)*	Percentage (%)
Certain herbal supplements	11	22.44
Custom herbal tea mixture	3	6.12
*Matricaria recutita*	4	8.16
*Withania somnifera*	3	6.12
*Silybum marianum*	2	4.08
*Curcuma longa*	2	4.08
*Urtica dioica*	2	4.08
*Valeriana officinalis*	2	4.08
Other monoherbs	16	32.65

*Primary reason for applying phytomedicines*		
	*n *= 48	Percentage (%)
General well-being	13	27.08
Acute disease	20	41.67
Chronic disease	6	12.50
Prevention	10	20.83

*Subjective evaluation of perceived benefit*		
	*n *= 47	Percentage (%)
Very useful	32	68.09
Moderately useful	14	29.79
I do not know	1	2.13

Categorizing the motivations behind the use of phytomedicines the authors found that acute disease is the most common reason, cited by 41.67% of respondents (Table 6). This suggests that phytomedicines are frequently used for short-term or sudden health issues. General well-being is the second most common reason (27.08%), indicating a significant use of phytomedicines for holistic or non-specific health maintenance. In using phytomedicines, prevention accounts for 20.83%, showing a proactive approach to health. Chronic disease is the least cited reason (12.50%), which may reflect either limited perceived efficacy for long-term conditions or a preference for conventional treatments in such cases. It might warrant further investigation into either the efficacy or awareness of phytomedicinal options for such conditions. Reflecting users’ personal assessments of the effectiveness of phytomedicines, a large majority (68.09%) found them very useful, indicating high satisfaction (Table 6). A proportion of 29.79% of respondents rated the therapies as moderately useful, suggesting some benefit but perhaps not consistently strong effects. Only 2.13% were uncertain (“I do not know”), showing that most users had formed a clear opinion on their effectiveness. The high satisfaction rate supports the potential value of phytomedicines in complementary or alternative medicine, though subjective evaluations should ideally be supplemented with clinical outcomes for a more robust assessment.

Only two respondents (4.08%) indicated the use of homeopathic medicines in the questionnaire. One of them uses an over-the-counter homeopathic mixture (composition: 0.375 mg each of *Cocculus indicus* 4 C, *Nux vomica* 4 C, *Tabacum* 4 C, *Petroleum rectificatum* 4 C), while the other takes three identified medicines (0.01 ml *Anas barbariae hepatis et cordis extractum* 200 K, *Rhus toxicodendron*,* Arnica montana* - the doses of the latter two are unknown). According to self-reporting, these medicines are used exclusively for acute complaints. Based on efficacy, the first participant found the mixed preparation somewhat useful, whereas the other considered the three medicines particularly beneficial for acute complaints. These data reflect that generally healthy respondents do not typically resort to homeopathic medicines, and if they do, it is only for acute complaints, in contrast to herbal products or vitamins. The high contrast between the widespread, highly satisfactory use of phytomedicines and the marginal role of homeopathy is particularly revealing. It suggests that users make a clear, pragmatic distinction between different “natural” therapies. Phytomedicines, with their detectable active ingredients, are embraced for both acute issues and well-being, aligning with an evidence-informed model of self-care. Homeopathy, based on a different principle, remains a niche choice for a very small subset, even within a CAM-using population. This pattern challenges the notion of CAM as a homogeneous field and indicates that public adoption is heavily influenced by perceived plausibility and tangible results, creating a natural hierarchy even among alternative therapies.

Supplements containing minerals and vitamins are considered as forms of orthomolecular medicine, aiming to restore and maintain optimal health by correcting nutritional imbalances and supporting physiological functions at the molecular level. Among ophthalmologically healthy volunteer respondents, the highest proportion (71.43%) reported consuming these products, and for 91.43% of them, such supplements still form part of their daily diet (Table 7). The most frequently consumed supplements were Vitamin D (62.86%), Vitamin C (57.14%), Multivitamins (37.14%), Magnesium (20%), and Vitamin B complex (17.14%). According to self-reports, these supplements are primarily used for preventive health purposes (46.15%) and to maintain general physical well-being (38.46%). One participant indicated the use of Vitamin C specifically in the context of an acute illness (1.28%). The high satisfaction rates may explain the continued use of these supplements: 67.11% of respondents rated them as very useful. However, 15.79% were unable to assess their effectiveness, suggesting that some individuals may be taking them out of habit rather than for a clearly defined therapeutic purpose. One respondent reported that the supplement in question was not useful for them.

**Table 7:** Prevalence, current usage, types of orthomolecular supplements consumed, primary reasons for intake, and subjective evaluation of perceived benefits among vitamin and mineral supplement users (*n* = 35)

	*n*	%
Prevalence of vitamin and mineral supplement use (*n* = 49)	35	71.43
Currently active consumer:	32	91.43
*Applied orthomolecular supplements*		
Vitamin D	22	62.86
Vitamin C	20	57.14
Multivitamins	13	37.14
Magnesium	7	20.00
Vitamin B complex	6	17.14
Selenium	2	5.71
Vitamin E	2	5.71
Coenzime Q10	1	2.86
Folate	1	2.86
Iron	1	2.86
Magnesium + Vitamin B6	1	2.86
Vitamin A	1	2.86
Vitamin K2	1	2.86

*Primary reason for consuming orthomolecular supplements*	*n *= 78	%
General well-being	30	38.46
Acute disease	1	1.28
Chronic disease	11	14.10
Prevention	36	46.15

*Subjective evaluation of perceived benefit*	*n*= 76	%
Very useful	51	67.11
Moderately useful	12	15.79
I do not know	12	15.79
Not useful at all	1	1.32

Based on self-reports from the questionnaire respondents, 26.53% consumed dietary supplements that could not be classified into the predefined categories. Notably, the highest proportion of these individuals (81.71%) are still currently using such products (Table 8). It is important to note that this categorization is arbitrary; the listed products were those that participants themselves could not assign to the categories of herbal, homeopathic, or vitamin and mineral supplements. Some respondents included herbal supplements in this category (19.05%), particularly those who considered tea preparations to be plant-based, while others classified similar products in the phytomedicine category. Hair vitamins, although compositionally similar to the previously listed orthomolecular products, were not perceived as “simple” multivitamins by participants. This section of the survey, therefore, lacks consistency, which is not due to issues with the Hungarian translation, but rather reflects a broader challenge related to health literacy—specifically, the general public’s limited familiarity with the official classification of health products available on the market.

**Table 8:** Summary of the other non-categorized supplements consumed by the survey respondents

	*n*	%
*Prevalence of non-categorized supplements* (*n* = 49)	13	26.53
*Currently active consumer*:	12	24.49
*Consumption of additional non-categorized supplements*		
*Currently applied*	18	85.71
Collagen	5	23.81
Herbal supplements	4	19.05
Hair vitamins	3	14.29
Protein	2	9.52
Spirulina	2	9.52
Fish oil (and omega fatty acids)	2	9.52
Humic acid	1	4.76
Hyaluronic acid + Collagen	1	4.76
Creatine	1	4.76

*Primary reason for consuming additional supplements*	*n = 25*	%
General well-being	9	36.00
Acute disease	3	12.00
Chronic disease	4	16.00
Prevention	9	36.00

*Subjective evaluation of perceived benefit*	*n = 27*	%
Very useful	19	70.37
Moderately useful	8	29.63
I do not know	0	0.00
Not useful at all	0	0.00

Excluding plant-based products, the most frequently purchased supplements were collagen (23.81%) and hair vitamins (14.29%) (Table 8). Notably, all three respondents who reported using hair vitamins were female. Protein powders, spirulina algae, and unsaturated fatty acids were consumed at similar rates (9.52% each). Similar to the pattern observed with orthomolecular supplements, the majority of respondents (72%) reported using these products primarily for general physical well-being and disease prevention. Satisfaction levels were high: 70.37% rated the products as very useful, and 29.63% as moderately useful. No negative or neutral responses were recorded.

Considering all responses related to dietary supplement use, it can be concluded that ophthalmologically healthy volunteers consume over-the-counter products, primarily vitamins, minerals, and collagen powders, regardless of the presence of illness, with the aim of maintaining health and enhancing general well-being. In contrast, for acute conditions, monoherbal teas are preferred for specific therapeutic purposes. Homeopathic preparations were notably underrepresented in this small sample, and when used, they were also associated with acute complaints. The near-universal adoption and high satisfaction with vitamin and mineral supplements among this health-conscious cohort point to their status as a foundational pillar of modern preventative self-care. However, this widespread use, particularly for non-specific goals like “general well-being,” raises a critical question: are these supplements primarily driven by evidenced-based nutritional needs or by powerful marketing and a cultural trend of “over-supplementation”. The fact that 15.79% of users could not assess their effectiveness is telling; it suggests that for a significant minority, consumption is a ritual of wellness rather than a targeted therapeutic intervention. This behavior aligns more with a proactive health identity than with a direct response to a diagnosed deficiency, highlighting a potential gap between public practice and clinical necessity. The use of herbal and mineral-based dietary supplements was widespread among participants, with no significant differences observed across gender or age groups (*p* > 0.05). Individuals frequently consumed multiple different preparations, indicating a generalized, non-specific pattern of use. This homogeneity likely reflects a modern, proactive approach to health and prevention. Unlike provider-based therapies, which are often sought for specific ailments and are influenced by cultural perceptions, supplements are easily accessible and marketed as foundational elements of daily wellness. Their consumption appears to be driven by a broad-based health consciousness and a preventative mindset that is adopted uniformly across demographics.

#### Self-help practices

Participants reported engaging in at least one of the listed self-help practices for personal health, including meditation, yoga, qigong, tai ji, relaxation techniques, visualization, traditional healing ceremonies, and prayer. These practices were frequently incorporated into daily or weekly routines. Meditation and relaxation techniques were each reported by 13 participants, with 61.5% and 69.2%, respectively, citing general well-being as their primary motivation (Table 9). Notably, 92.3% of participants rated meditation as very useful, while 7.7% considered it moderately useful. Relaxation techniques were similarly well-received, though rated slightly less favorably, indicating overall high satisfaction with both methods. Yoga was practiced by eight participants, with 50% reporting general well-being and the other 50% citing chronic health conditions as their main reasons for use. Visualization (*n* = 8) and prayer for personal health (*n* = 7) were also commonly practiced, with all participants (100%) identifying general well-being as their primary motivation. In the responses, personal prayer was consistently reported as a general practice for well-being and was not linked to specific health conditions. Within the Hungarian cultural landscape, it can therefore be interpreted as an expression of broad religiosity rather than a targeted therapeutic intervention. Qigong and tai ji were each reported by only one participant from the older than 35-year-old cohort, suggesting that mind-body practices of TCM are not widely adopted among ophthalmologically healthy individuals in this sample. A clear generational divide was observed in the practice of certain mind-body and traditional therapies. According to self-reports, none of the participants under the age of 35 were engaged in Taiji, Qigong, or traditional healing rites. In contrast, these activities were exclusively reported by older participants, with three individuals from the ≥ 35 age group participating in traditional healing. This stark discrepancy suggests that these specific practices are perceived differently across generations. For younger individuals, they may be viewed as less relevant or accessible compared to more modern wellness activities like meditation or yoga. Their adoption by older adults could be influenced by a stronger connection to cultural traditions, a different approach to managing age-related health concerns, or simply greater lifetime exposure to these specific practices. This indicates that the acceptance of certain CAM modalities is not only a matter of personal choice but is also significantly shaped by generational and cultural context.

**Table 9:** Reported use, primary reasons for engagement, and subjective evaluation of perceived benefit from various mind-body and spiritual practices among participants (*n* = 56)

	Meditation		Yoga		Qigong		Taiji		Relaxation techniques		Visualization		Traditional healing ceremony		Praying for own health		Other^a^	
Practices reported by (*n* = 56)	*n* = 13	23,2%	*n* = 8	14,3%	*n* = 1	1,8%	*n* = 1	1,8%	*n* = 13	23,2%	*n* = 8	14,3%	*n* = 3	5,4%	*n* = 7	10,7%	*n* = 3	5,4%
*Primary reason for consulting healthcare providers*																		
General well-being	8	61.5	4	50.0	1	100.0	1	100.0	9	69.2	3	37.5	1	33.3	3	42.9	2	66.7
Acute disease	1	7.7	0	0.0	0	0.0	0	0.0	0	0.0	0	0.0	0	0.0	0	0.0	0	0.0
Chronic disease	4	30.8	4	50.0	0	0.0	0	0.0	3	23.1	4	50.0	2	66.7	3	42.9	1	33.3
Other^b^	0	0.0	0	0.0	0	0.0	0	0.0	1	7.7	1	12.5	0	0.0	1	14.3	0	0.0
*Subjective evaluation of perceived benefit from healthcare services*																		
Very useful	12	92.3	7	87.5	1	100.0	1	100.0	11	84.6	8	100.0	3	100.0	7	100.0	3	100.0
Moderately useful	1	7.7	0	0.0	0	0.0	0	0.0	2	15.4	0	0.0	0	0.0	0	0.0	0	0.0
I do not know	0	0.0	0	0.0	0	0.0	0	0.0	0	0.0	0	0.0	0	0.0	0	0.0	0	0.0
Not useful at all	0	0.0	1	12.5	0	0.0	0	0.0	0	0.0	0	0.0	0	0.0	0	0.0	0	0.0

^a^Reported other practices: Tibetan singing bowl therapy (by 1 respondent), trekking and walking (by 1 respondent)

^b^A thorough explanation was not provided

The “Other” category (*n* = 3) included diverse practices such as Tibetan singing bowl therapy, trekking, and walking, with 66.7% motivated by general well-being and all participants reporting high levels of satisfaction. None of the participants reported using these practices primarily for acute conditions, with the exception of one participant who used meditation for an acute issue. This suggests that such self-help practices are predominantly utilized for chronic conditions or general mind-body wellness. Overall, subjective evaluations of benefit were largely positive, with yoga receiving a single report of being not useful.

Fisher’s exact tests revealed no statistically significant associations between gender and the application of any specific CAM therapy. The utilization rates for meditation (*p* = 0.759), yoga (*p* = 0.706), relaxation techniques (*p* = 0.524), visualization (*p* = 0.706), prayer (*p* = 0.686), and participation in traditional healing ceremonies (*p* = 0.607) were all independent of gender. Although minor numerical differences were observed in some groups, for instance, a slightly higher percentage of men reported using relaxation techniques (54%) compared to women (46%), none of these differences reached statistical significance. This indicates that the use of these mind-body and spiritual therapies was not gender-specific within the studied cohort.

Application of most CAM therapies was independent of age group, as the exact tests revealed in this case. No statistically significant associations were found between age (< 35 years vs. ≥35 years) and the use of meditation (*p* = 0.714), yoga (*p* = 0.675), relaxation techniques (*p* = 0.441), or prayer (*p* = 0.619). However, a significant association was identified for the use of visualization techniques (*p* = 0.044). Participants under 35 years of age were significantly more likely to apply visualization (50%, 4/8 of all users) compared to their older counterparts, despite the younger cohort constituting only 22% of the total sample. This indicates that while the utilization of most mind-body therapies was similar across age groups, visualization was distinctly more prevalent among younger participants. The significant association may be explained by several factors. Younger individuals may exhibit greater openness to innovative or mind-based complementary approaches. Furthermore, this finding could reflect generational differences in health-seeking behaviors or age-specific preferences for certain stress-management strategies. This result is particularly notable as it represents the only significant age-based difference identified across all CAM therapies analyzed in this study.

The survey results included responses to a question asking participants to share their opinions or suggestions regarding the overall questionnaire or the topic in question. The feedback is summarized as follows: one respondent mentioned that sharing personal experiences could be beneficial in the future, indicating a desire for more qualitative input in the survey process. Another respondent simply found the survey useful, suggesting that the content was relevant and informative. A third respondent appreciated the topic but felt that the questionnaire was somewhat lengthy, highlighting a potential area for improvement in terms of survey design and respondent engagement. Lastly, one respondent suggested focusing on improving quality of life rather than just well-being, which could imply a broader perspective on health and wellness topics. This feedback can be instrumental in refining future surveys to better meet the needs and expectations of respondents.

## Discussion

According to the WHO, CAM encompasses a broad range of health care practices that are not part of a country’s conventional medical system and are not fully integrated into mainstream health care, yet therapies such as acupuncture, herbal medicine, homeopathy, and chiropractic care are becoming increasingly popular in Western societies, with usage patterns varying across individuals and nations [4]. The self-reported use of CAM varies from 10% to 76% worldwide [3]. The results indicate that CAM is widely used, particularly in the context of herbal therapies and vitamins [14]. The reasons for using CAM therapies vary by country. Numerous statistics are available regarding the global use of CAM; however, the heterogeneity of survey methodologies makes it difficult to obtain precise and comparable data [21]. A 2002 survey reported that 36–42% of the adult population in the United States utilized CAM services, while more recent statistics suggest this figure has risen to 72% [21]. In Europe, a 2013 survey indicated that 56% of the population preferred CAM therapies [21]. In Hungary, the most recent nationally representative study was conducted in 1999, revealing that 23.1% of the adult population had used CAM at least once [21]. Since then, no similar large-scale survey has been carried out. The I-CAM-Q questionnaire, developed by Quandt et al., aims to address these challenges by providing a standardized, internationally applicable, and easily translatable tool for assessing CAM use [1]. In the present study, this questionnaire was validated in Hungarian. The data provided by the I-CAM-Q has contributed to understanding the CAM usage patterns of different populations and the sociocultural factors associated with them.

The long-term objective of the authors in adapting and validating the I-CAM-Q questionnaire into Hungarian is to explore the attitudes and interest of ophthalmic patients in Hungary toward complementary medicine. As a preliminary step, the survey was pilot-tested on a small sample of Hungarian-speaking volunteers with no significant ophthalmological conditions. This initial phase was successfully completed. Although the literature on this topic is limited, existing studies support the authors’ conceptual framework. Recent studies highlight a growing global interest in complementary therapies among ophthalmic patients. In the Middle East, reports from 2021 indicate that between 22% and 67% of patients utilized such therapies, particularly in connection with eye conditions. Despite this, awareness regarding scientific evidence and potential adverse effects remains limited [16]. A 2010 Canadian survey revealed that 13.6% of glaucoma patients used complementary treatments alongside conventional antiglaucoma medications, and 62.5% did not disclose this use to their ophthalmologists. Among the therapies employed, herbal remedies (34.5%), dietary changes (22.7%), and nutritional supplements (18.8%) were the most common [16]. While 40.5% of users were satisfied, 19.2% were uncertain about the efficacy of these interventions. Notably, only 16.7% received information from their ophthalmologist, whereas media sources accounted for 37.1% of the information dissemination [16, 22]. A 2014 follow-up study focusing on Canadian ophthalmologists found that younger practitioners (under 50 years of age, with less than 20 years of experience) were more likely to inquire about patients’ use of complementary therapies (26%) and to recommend them (9%) [23]. The most frequently endorsed approaches included regular exercise (28%), *Ginkgo biloba* L. (Ginkgoaceae) supplementation (21%), antioxidant vitamins (16%), and a diet low in fat and sodium but rich in fruits and vegetables (12%) [23]. Collectively, these findings suggest a global trend toward an increased utilization of complementary therapies in ophthalmology, potentially driven by the chronic and age-related nature of many eye diseases. A review by Robinson & McGrail of an Australian patient population found that disclosure of CAM use was highly dependent on the physician’s familiarity with such therapies. The disclosure rate was highest (90%) when the practitioner also practiced a form of CAM but fell to as low as 12% when the practitioner was unfamiliar with them. The primary reasons for non-disclosure were the fear of a negative response, including potential prejudice or discontinuation of therapy, as well as the perception of physician incompetence, skepticism, or disinterest [24].

### Discussion on healthcare services

Based on the results of the present Hungarian questionnaire, the vast majority of ophthalmologically healthy volunteers would primarily consult a physician, yet they would also consider using CAM therapies alongside evidence-based treatments, provided they are granted autonomy in therapeutic decision-making. In the hypothetical event of an ophthalmic condition, however, they would be less inclined to inform their ophthalmologist about CAM use, either due to perceiving ophthalmologists as lacking expertise in this area or not feeling the need to disclose such information. Furthermore, participants indicated a greater willingness to use CAM therapies if the associated costs represented minimal burden on their income.

In a Swedish study conducted by Wemrell et al. involving 1534 participants, 88% reported having consulted a conventional healthcare provider within the past year [8]. 70% of respondents indicated that they had used both conventional medicine and CAM, while 28% reported exclusive use of conventional medical services [8]. In the Hungarian sample the authors did not report any informant with an exceptional preference of a particular CAM provider. Among CAM modalities, manual therapies were particularly prevalent: 52.5% of respondents reported using massage therapy, 17.0% had visited a chiropractor, and 11.3% had received naprapathic treatment (a special body manipulation and lifestyle therapy of Czech origin) [8]. These practices reflect a strong emphasis on physical manipulation and bodywork aimed at improving physical well-being and musculoskeletal conditions. In a Norwegian study by Kristoffersen et al. (*n* = 385), 62.2% of the respondents reported using some form of CAM within the previous 12 months. During the same period, 76.1% consulted a physician, while 10.2% visited a chiropractor and 7.4% a massage therapist. For acute health conditions, chiropractors (33.2%), physicians (25.5%), and naprapaths (23.2%) were the most frequently consulted providers. Regarding CAM modalities used for general well-being, 55.3% of respondents reported visiting a massage therapist, 49.1% a reflexologist (this therapy method was not reported in the Hungarian sample), and 39.7% consulted a phytotherapist. Across all categories’, perceived helpfulness was remarkably high, ranging from 85% to 100%, with kinesiologists and traditional healers receiving the highest ratings (100%). Only 2.32% of CAM services were provided by physicians, yet satisfaction with these services was notably high (76.4%–100%). In contrast, the use of non-medically regulated CAM modalities such as acupuncture, and massage were low (0.6% each) [3]. In a study conducted by Jędrzejewska et al. (*n* = 524), the majority of Polish respondents reported consulting a physician primarily for chronic conditions (57.5%), while only 16.2% sought medical care for acute illnesses [4]. Among CAM providers, 27.3% visited a phytotherapist, 25.2% consulted other unspecified specialists, and 20.8% used acupuncture [4]. Other specialists (81.8%), spiritual healers (81.2%), and homeopaths (78.5%) were reported to have the highest perceived benefit [4]. Similar to findings from the Hungarian sample, naturopathic therapies, particularly manual therapies provided by doctors were commonly used, (27.1%) and herbalism (21.6%), with homeopathy (10.1%) and cupping (11.1%) used at comparable rates. Satisfaction with naturopathic treatments administered by physicians was generally high (58.6%–70.0%), with very low levels of dissatisfaction [4]. In a large-scale Japanese study by Motoo et al. (*n* = 3208), 12.8% of randomly selected healthy respondents reported using CAM within the past 12 months [13]. A distinctive feature of the Japanese sample, compared to European populations, is the integration of Japan’s own traditional medical system (Kampo medicine), which includes unique products and practices. In terms of conventional healthcare, 54.8% of respondents consulted physicians, while 34.9% visited dentists, 29.4% pharmacists, and 18.5% nurses [13]. In the Hungarian survey pharmacist was also reported by 1 informant. The most commonly used CAM modalities by the Japanese population included over-the-counter Kampo medicines (15.7%) and prescribed Kampo medicines (15.4%), followed by dietary supplements (11.8%), massage services (3.9%), and physical therapy (3.5%) [13]. CAM practitioners consulted included massage and shiatsu therapists (5.7%), physical therapists (4.9%), judo therapists (3.1%), and acupuncture/moxibustion practitioners (2.6%) [13]. In the study conducted by Tabata et al. (*n* = 164), the majority of participants (43.3%) reported consulting a physician for health-related concerns. This was followed by consultations with massage therapists (17.7%), acupuncture and moxibustion practitioners (12.2%), and judo therapists (11.0%) [12].

### Discussion on supplements and medicines

In the survey of Harnett et al. vitamins and minerals were the most frequently recommended by healthcare providers, and herbal supplements were only in second place [25]. The same was observable in the Hungarian pilot survey respondents. Herbal medications (excluding homeopathic preparations) have become increasingly prevalent all over the world, and people are increasingly accepting them [16]. A noteworthy finding is the low prevalence of homeopathic medicines within the studied group. Historically, from the 1840 s, Hungary—as part of the Austro-Hungarian Empire—was a stronghold of homeopathic hospitals and treatments [26]. In contrast, today, the majority of the population does not consider homeopathy to be a rational form of naturopathic therapy [27]. According to Hungarian legislation, the practice of homeopathy is restricted to individuals holding a medical degree. Specifically, “Government Decree 40/1997 (III.5)” and “Decree 11/1997 (V.28) of the Ministry of Social Welfare” stipulate that only medical doctors are permitted to practice homeopathy, as well as other forms of CAM such as anthroposophic medicine, TCM, and manual medicine [28, 29]. This regulation makes Hungary one of the stricter European Union member states in this regard, as in several other countries - such as the United Kingdom, and Germany - non-medically qualified practitioners may legally or tolerably practice homeopathy. There are countries such as Croatia and Slovenia, where medical doctors are prohibited from practicing homeopathy [17, 30]. The prevalence of homeopathic practice widely varies among European countries between 1% to ≥ 10% [27]. Kristoffersen et al. presented only 0.3% of Norwegian respondents applied homeopathic remedies [3]. Among the Japanese informants, Motoo et al. reported only 1 participant prescribed homeopathic medicine, which means a similar underrepresentation of this therapy in the Japanese population.

Among Swedish participants, 68.5% reported using nutritional supplements, while 25.3% indicated the use of herbal medicines [8]. Two-thirds of Norwegian respondents (68.1%) reported using natural remedies and 51.1% used vitamins and minerals. The most commonly used products included cod-liver oil (19%), vitamin D (18%), multivitamins (16%), omega-3 fatty acids (11%), vitamin C (10%), magnesium (8%), B vitamin complex (8%), iron (4%), calcium (3%), and *Vaccinium myrtillus* L. (Ericaceae) extract (1%) [3]. Calcium supplementation was not reported by the Hungarian informants. Polish respondents also reported high levels of supplement use; mirroring the trends observed in Hungary. Orthomolecular supplement use was reported by 88.4%, and herbal medicine use by 84.7%. Notably, satisfaction was slightly higher for homeopathic remedies and other non-categorized supplements (74.0%–79.6%) [4]. Regarding specific substances, the most frequently used single herbs among the Polish survey respondents included *Urtica dioica* L. (Urticaceae), *Melissa officinalis* L. (Lamiaceae), *Withania somnifera* (L.) Dunal (Solanaceae). *Mentha × piperita* L. (Lamiaceae), *Cannabis sativa* L. (Cannabaceae), *Morus alba* L. (Moraceae), *Silybum marianum* (L.) Gaertn. (Asteraceae), and *Matricaria recutita* L. (Asteraceae) [4]. These herbs were mostly similarly reported by the Hungarian volunteers except *M. alba*. The most commonly used vitamins and minerals were vitamin D3, vitamin C, magnesium, and B-complex vitamins [4], which is consistent with the Scandinavian and Hungarian samples. Among vitamin-based CAM products, vitamin C was the most frequently used supplement (10.6%), followed by multivitamins (7.9%), reported Motoo et al. The most commonly used minerals were calcium (6.3%), iron (6.0%), and zinc (5.1%) [13]. Regarding herbal supplements, *Vaccinium myrtillus* L. (Ericaceae) was used by 8.9% of respondents, and “green juice” by 7.5% [13]. Notably, while *V. myrtillus* was mentioned less frequently in Norwegian studies, it was not reported at all among Hungarian respondents.

### Discussion on self-help practices

Mind-body practices were also represented in the Swedish population, with 26.3% engaging in breathing techniques, 20.2% practicing yoga, and 16.5% using relaxation techniques [8]. Among self-help practices, yoga (14.8%), meditation or mindfulness (12.2%), relaxation techniques (9.8%), prayer (4.2%), and visualization (3.8%) were the most frequently used methods by Norwegians. The majority of the respondents rated these practices as helpful (84%–100%), with women engaging in self-help modalities more frequently than men [3]. In terms of self-help practices, the most frequently reported methods were relaxation techniques (49.6%), prayer for personal health (43.3%), and meditation (41.2%). Less commonly used practices included participation in traditional healing ceremonies (3.1%), Tai ji (4.4%), and Qigong (5.9%). The primary motivation for engaging in these practices was to improve overall well-being [4]. The Polish sample shows a slightly higher number of participants in TCM practices than the Hungarian respondents, however, the religious prayer is similarly important. Self-care practices were reported by 47.9% of Japanese participants in the past year. The most commonly used methods included bath salts (25.8%) and walking (25.3%) [13]. The average frequency of use over a three-month period was highest for praying for health (36.2 times), followed by bath salts (28.2 times) and music therapy (26.7 times). Walking was perceived as helpful by 89.4% of respondents, with 23.6% rating it as very helpful and 65.8% as somewhat helpful [13]. Interestingly, daily prayer for health was also commonly reported among Hungarian respondents. In contrast, only one Hungarian respondent in the present study mentioned trekking or walking as a form of self-help, suggesting that, compared to Japanese participants, healthy Hungarian individuals may be less inclined to engage in physical activity as a therapeutic strategy. A significant proportion of the Japanese participants (93.8%) reported practicing traditional East Asian self-care methods such as yoga, Tai Ji, and Qigong to enhance their overall well-being. Additionally, 90.0% used hot-spring therapy, 82.9% practiced dietary therapy, 78.0% engaged in exercise therapy, 75.0% used aromatherapy, 72.2% practiced Zen or meditation, and 66.7% reported praying for health [12]. When asked about the perceived effectiveness of these practices, more than half of the participants rated yoga, Tai Ji, Qigong, dietary therapy, aromatherapy, and meditation as “very helpful” [12]. While prayer was also a common and valued practice among Hungarian respondents, modalities such as yoga, Tai Ji, and Qigong—deeply rooted in East Asian cultural traditions—remain less familiar and less frequently practiced as self-help strategies in Western contexts, including Hungary. Aromatherapy is a globally growing trend of CAM therapy [16], however only the Japanese surveys reported its significant use between 0.7% and 22% improving well-being (75–84.6.6%) with moderate level of satisfaction (27.9–52.8%) [12, 13]. Unfortunately, the Hungarian sample did not mention essential oils or aromatherapy as self-help method.

To ensure linguistic and conceptual equivalence, the I-CAM-Q questionnaire was translated into Hungarian using a forward-backward translation process, which involved independent bilingual translators and reconciliation by an expert panel, thereby enhancing the accuracy and cultural relevance of the adapted version. The questionnaires were conducted with healthy volunteers to assess the clarity, comprehensibility, and cultural appropriateness of the translated items, allowing for refinement of the questionnaire and supporting its reliability in the Hungarian context. Güthlin et al. reported in their pilot validation of the German version of the questionnaire, conducted with 16 healthy participants, that the original instrument lacked written instructions. As a result, a set of instructions was developed to ensure clarity and comprehensibility for respondents [7, 31]. In contrast, the Hungarian version did not require additional written guidance, as the questions were already well-adapted to familiar Hungarian terminology and were easily understood by the participants. However, some respondents found the overall length of the survey burdensome and time-consuming. Small pilot sample size can be mentioned as the main limitation of the study. Although the Hungarian sample was not the smallest pilot I-CAM-Q group mentioned in the literature, it was larger than those in the Argentinian (*n* = 18) [15], German (*n* = 16) [7, 31], and French (*n* = 10) [5] validation and adaptation pilot studies. Furthermore, the questionnaire in its current form does not comprehensively assess respondents’ belief systems, prior experiences, health literacy, or their emotional and cognitive attitudes toward CAM therapies. These psychometric dimensions are specifically addressed by the Perspectives on the Use in Communities of CAM Questionnaire (PUC-CAM-Q), developed by Robinson et al., which has shown promise as a tool for exploring CAM use at the community level [32]. The conceptual framework of the PUC-CAM-Q may prove valuable in future studies involving Hungarian-speaking populations, particularly given that a similar literature review has already been conducted in Hungary by Zörgő et al. [21].

The successful adaptation of the I-CAM-Q into Hungarian is a significant achievement, as it provides a standardized tool for assessing CAM use in Hungary. The translation process was rigorous, involving multiple stages of review and testing to ensure accuracy and cultural relevance. The pre-testing phase confirmed that the Hungarian version was well-understood by participants, with no significant linguistic or technical barriers encountered. This adaptation allows for reliable data collection on CAM use in Hungary, contributing to a better understanding of CAM practices and their integration into the healthcare system. The availability of a validated Hungarian version of the I-CAM-Q will facilitate research on CAM use in Hungary, enabling comparisons with data from other countries and contributing to the global understanding of CAM practices. Furthermore, the data collected using the Hungarian I-CAM-Q can inform healthcare policies and practices, leading to improved patient care and outcomes.

This study has some limitations that should be considered. The sample size was relatively small (*n* = 49) and was primarily composed of healthcare workers of ophthalmology departments. While this group was highly suitable for the initial pilot testing of our methodology, it represents a convenience sample that lacks diversity. As a result, the findings may be influenced by the specific expertise and biases inherent to this professional group, thereby limiting their applicability to the general population. Further research should aim to include larger, more diverse samples to validate and extend these preliminary results.

Furthermore, a formal psychometric evaluation was not conducted as part of this initial adaptation and validation study. The primary goal of this phase was to establish the linguistic and conceptual equivalence of the Hungarian I-CAM-Q and to conduct a preliminary assessment of its feasibility and comprehensibility in a general population sample. This approach is consistent with several other I-CAM-Q validation studies (e.g. the Polish version of I-CAM-Q elaborated by Jędrzejewska et al. [4]) that prioritized cross-cultural comparability through rigorous translation over immediate psychometric testing in the first phase. The nature of the I-CAM-Q as a descriptive, utilization-focused tool, rather than a psychological scale, means that traditional reliability metrics like internal consistency are less critical. Our aim for the next phase of research is to extend the study to ophthalmic patient groups and perform a full psychometric validation, thereby establishing the instrument’s reliability and validity in that specific clinical context.

## Conclusion

The successful adaptation and validation of the I-CAM-Q for the Hungarian context with supplementation of general demographics and ophthalmology questions provides a valuable tool for assessing the use of complementary and alternative medicine in Hungary. However, the true value of this research lies not merely in the instrument itself, but in its potential to catalyze critical advancements in Hungarian healthcare policy and education. The primary implication of our findings is the urgent need to integrate structured CAM modules into the Hungarian medical curriculum. Educating future physicians about prevalent CAM practices is crucial for fostering open doctor-patient communication and ensuring safe, coordinated care. Future studies should focus on further validating the psychometric properties of the questionnaire and exploring its use in diverse populations within Hungary. Additionally, longitudinal studies could be conducted to assess changes in CAM use over time and to evaluate the impact of CAM practices on health outcomes. Documenting CAM use as a routine part of clinical practice would significantly enhance pharmacovigilance, illuminate potential herb-drug interactions, and provide a more holistic view of the patients’ health-seeking behavior. To implement these changes effectively, Hungary should pursue collaboration with established European CAM networks, such as the NAFKAM model. Such partnerships would accelerate the development of evidence-based guidelines, support professional training, and integrate Hungary as an active participant in the European dialogue on integrative medicine. In summary, the successful adaptation of the I-CAM-Q into Hungarian represents a significant step toward a more informed, transparent, and integrative healthcare system in Hungary.

## Supplementary Information

Supplementary Material 1.

Supplementary Material 2.

Supplementary Material 3.

## Data Availability

The datasets used and/or analyzed during the current study are available from the first author on reasonable request.

## Abbreviations

AMD: Age-related macular degeneration
BCVA: Best corrected visual acuity
CAM: Complementary and Alternative Medicine
I-CAM-Q: International Complementary and Alternative Medicine Questionnaire
NAFKAM: Norway's National Research Centre for Complementary and Alternative Medicine (Norsk Senter for Alternativ og Komplementær Medisin)
PUC-CAM-Q: Perspectives on the Use in Communities of CAM Questionnaire
TCM: Traditional Chinese Medicine
