# Supplementary material for: Translation and cultural adaptation of the I-CAM-Q: the first Hungarian version for assessing complementary and alternative medicine use
Source: BMC Complement Med Ther. 2025 Dec 20;26:24. doi: 10.1186/s12906-025-05220-2 (PMC12837113; doi:10.1186/s12906-025-05220-2)
Supplement: Supplementary file 1 — Supplementary Material 1. [file 12906_2025_5220_MOESM1_ESM.docx]

**Melléklet:**

**Demográfiai kérdések**

1. Biológiai neme:

- Férfi
- Nő
  - - Nem kívánok válaszolni

1. Melyik korcsoportba tartozik?

- 18-24 év
- 25-34 év
- 35-44 év
- 45-54 év
- 55-64 év
- 65 év felett

2. Végzettsége:

- Általános iskola
- Középiskola
- Felsőfokú végzettség (főiskola/egyetem)
- Posztgraduális végzettség

1. Jövedelmi kategória:

- 0-500 000 Ft
- 500 000-1 000 000 Ft
- 1 000 000 Ft felett

1. Lakhely (Budapest vagy vármegye):

- Budapest
- Bács-Kiskun
- Baranya
- Békés
- Borsod-Abaúj-Zemplén
- Csongrád-Csanád
- Fejér
- Győr-Moson-Sopron
- Hajdú-Bihar
- Heves
- Jász-Nagykun-Szolnok
- Komárom-Esztergom
- Nógrád
- Pest
- Somogy
- Szabolcs-Szatmár-Bereg
- Tolna
- Vas
- Veszprém
- Zala

1. Önértékelt egészségi állapot:

- Kiváló
- Nagyon jó
- Jó
- Közepes
- Rossz

1. Legalább egy ismert betegségem van:

- Igen
- Nem

1. Ismert betegség részletezve – amennyiben *Igen*nel válaszolt (Több válasz is lehetséges):

- Magas vérnyomás
- Cukorbetegség
- Asztma
- Daganatos kórképek
- Egyéb: …………………………………………………………………………

1. Ismert szembetegség (Több válasz is lehetséges):

- Szürkehályog
- Zöldhályog
- Diabéteszes retinopátia
- Időskori maculadegeneráció (AMD)
- Szemüveggel javítható állapotok
- Száraz szem
- Egyéb: …………………………………………………………………………
- Nincs ismert szembetegségem

1. Családban ismert szembetegség (Több válasz is lehetséges):

- Szürkehályog
- Zöldhályog
- Diabéteszes retinopátia
- Időskori maculadegeneráció (AMD)
- Szemüveggel javítható állapotok
- Száraz szem
- Egyéb: …………………………………………………………………………
- Nem tudok a családban ismert szembetegségről

1. Hallott már valaha alternatív kezelésekről a szemészeti betegségek esetén?

- Igen
- Nem

1. A komplementer (kiegészítő és alternatív) terápiákat illetően honnan szerzi információit? (Több válasz is lehetséges)

- Egészségügyi dolgozóktól
- Közösségi médiákból (pl. Facebook)
- Családi/ ismeretségi/ baráti körből
- Egyéb: …………………………………………………………………………
- Nem szerzek ilyen témában információt

1. A komplementer (kiegészítő és alternatív)terápiát mely szakembertől fogadna el? (Több válasz is lehetséges)

- Orvos végzettségűtől
- Nem orvos szakdolgozótól
- Egyéb egészségügyi végzettséggel nem rendelkezőtől
- Nem fogadnék el komplementer terápiát

1. Amennyiben kapott, megbeszélte kezelő szemészorvosával az Ön által alkalmazott komplementer (kiegészítő és alternatív) kezeléseket?

- Igen
- Nem, egyik kezelőorvosommal sem
- Szemészorvosommal nem, de más kezelőorvosommal igen

1. Amennyiben a „Nem” választ választotta, mi volt az oka?

- Félek a kezelőorvosom negatív véleményétől
- Attól tartok, hogy leállítaná a kezelést
- Nem érzem a kezelőorvosom kompetensnek a természetgyógyászati kezelésekben
- Egyéb ok: ……………………………………………………………………………….

1. Amennyiben kezelő szemészorvosa az Ön betegségére az orvostudományi bizonyítékokon alapuló gyógyszer/kezelés mellett kiegészítő/alternatív kezelést kínálna fel (pl. gyógynövényi hatóanyagot is tartalmazó szemcsepp az antibiotikumot tartalmazó szemcseppel szemben), melyiket fogadná el?

- A bizonyítékokon alapuló kezelést preferálnám
- Kiegészítő/alternatív kezelést preferálnám
- Mindkettő lehetőséget elfogadnám tájékoztatást és mérlegelést követően
- A kezelőorvosom szaktudására bíznám a választást

1. A komplementer (kiegészítő és alternatív) kezelésekre a havi fizetésének hány százalékát szánná, ha lenne rá módja?

- Fizetésemnek 10%-ánál kevesebbet.
- Nem számítana, ha magasabb lenne a havi költség, mint a fizetésem 10%-a.

*Kérjük, ossza meg véleményét vagy javaslatát a teljes kérdőíves felmérést, illetve a témát illetően:……………………………………………………………………………………………………………………………………………………………………………………………………………………………………………………………………………………………………………………….………………………………………………………………………………………………………………………………………………………….…………………………………………………………………………………………………………………………….. ………………………………………………………………………………………………………………………..*
