# Supplementary material for: Translation and cultural adaptation of the I-CAM-Q: the first Hungarian version for assessing complementary and alternative medicine use
Source: BMC Complement Med Ther. 2025 Dec 20;26:24. doi: 10.1186/s12906-025-05220-2 (PMC12837113; doi:10.1186/s12906-025-05220-2)
Supplement: Supplementary file 2 — Supplementary Material 2. [file 12906_2025_5220_MOESM2_ESM.docx]

***NAFKAM International CAM Questionnaire (I-CAM-Q):***

***RECOMMENDED FOR USE IN STUDIES OF***

***COMPLEMENTARY AND ALTERNATIVE MEDICINE (CAM) --***

***Self-Administered Version***

1. ***Visiting health care providers:***  Health problems may be attended to by a variety of complementary and conventional health care providers.

| Have you seen any of the following providers in the last 12 months? | Yes  No  Number of times you saw this provider in the last 3 months? | Please indicate the *main* reason you *last* saw the provider  (Check only *one*). | | | | How helpful was it for you to see this provider?  (Check only one) |
| --- | --- | --- | --- | --- | --- | --- |
|  |  | For an acute illness/condition, one that lasted less than one month | To treat a long-term health condition (one that lasted more than one month) or its symptoms | To improve well-being | Other  (Please specify the other reason) |  |
|  |  |  |  |  |  | Very  Somewhat  Not at all  Don’t know |
| **Physician** |  |  |  |  |  |  |
| **Chiropractor** |  |  |  |  |  |  |
| **Homeopath** |  |  |  |  |  |  |
| **Acupuncturist** |  |  |  |  |  |  |
| **Herbalist** |  |  |  |  |  |  |
| **Spiritual healer** |  |  |  |  |  |  |
| **Specified option:**  **____________** |  |  |  |  |  |  |
| **Other (please specify):**  **____________** |  |  |  |  |  |  |
| **Other (please specify):**  **____________** |  |  |  |  |  |  |

***2. Complementary treatments received from physicians (MDs)***

**If you have not seen a physician in the past 12 months, please go to question 3.**

Some physicians provide complementary, as well as conventional treatments

| Have you received any of the following complementary treatments from a physician in the last 12 months? | Yes  No  Number of times you received this treatment in the last 3 months? | Please indicate the *main* reason you *last* received this treatment  (Check only *one*). | | | | How helpful was it to receive treatment from the physician?  (Check only one) |
| --- | --- | --- | --- | --- | --- | --- |
|  |  | For an acute illness/condition, one that lasted less than one month | To treat a long-term health condition (one that lasted more than one month) or its symptoms | To improve well-being | Other  (Please specify the other reason) |  |
|  |  |  |  |  |  | Very  Somewhat  Not at all  Don’t know |
| **Manipulation** |  |  |  |  |  |  |
| **Homeopathy** |  |  |  |  |  |  |
| **Acupuncture** |  |  |  |  |  |  |
| **Herbs** |  |  |  |  |  |  |
| **Spiritual healing** |  |  |  |  |  |  |
| **Specified option:**  **____________** |  |  |  |  |  |  |
| **Other (please specify):**  **____________** |  |  |  |  |  |  |

***3. Use of Herbal Medicine and Dietary Supplements,*** including tablets, capsules and liquids.

| For each category below, please list up to three products you have used in the last 12 months. | Do you currently use this product? | Please indicate the *main* reason that applies to your *last* use  (Check only *one*). | | | | How helpful did you find this product?  (Check only one) |
| --- | --- | --- | --- | --- | --- | --- |
|  |  | For an acute illness/condition, one that lasted less than one month | To treat a long-term health condition (one that lasted more than one month) or its symptoms | To improve well-being | Other  (Please specify) |  |
|  |  |  |  |  |  | Very  Somewhat  Not at all  Don’t know |
|  | Yes  No |  |  |  |  |  |
| **Herbs/Herbal Medicine** | | | | | | |
| ________________ |  |  |  |  |  |  |
| ________________ |  |  |  |  |  |  |
| ________________ |  |  |  |  |  |  |
| **Vitamins/Minerals** | | | | | | |
| ________________ |  |  |  |  |  |  |
| ________________ |  |  |  |  |  |  |
| ________________ |  |  |  |  |  |  |
| **Homeopathic remedies** | | | | | | |
| ________________ |  |  |  |  |  |  |
| ________________ |  |  |  |  |  |  |
| ________________ |  |  |  |  |  |  |
| **Other Supplements** | | | | | | |
| ________________ |  |  |  |  |  |  |
| ________________ |  |  |  |  |  |  |
| ________________ |  |  |  |  |  |  |

***4. Self Help Practices***

| Have you used any of the following self-help practices in the last 12 months? | Yes  No  Number of times you used this practice in the last 3 months? | Please indicate the *main* reason that applies to your *last* use of the self-help practice (Check only *one*). | | | | How helpful did you find this self-help practice?  (Check only one) |
| --- | --- | --- | --- | --- | --- | --- |
|  |  | For an acute illness/condition, one that lasted less than one month | To treat a long-term health condition (one that lasted more than one month) or its symptoms | To improve well-being | Other  (Please specify the other reason) |  |
|  |  |  |  |  |  | Very  Somewhat  Not at all  Don’t know |
| **Meditation** |  |  |  |  |  |  |
| **Yoga** |  |  |  |  |  |  |
| **Qigong** |  |  |  |  |  |  |
| **Tai Chi** |  |  |  |  |  |  |
| **Relaxation techniques** |  |  |  |  |  |  |
| **Visualization** |  |  |  |  |  |  |
| **Attended traditional healing ceremony** |  |  |  |  |  |  |
| **Praying for own health** |  |  |  |  |  |  |
| **Specified option:**  **____________** |  |  |  |  |  |  |
| **Other (please specify):**  **____________** |  |  |  |  |  |  |

Source: Quandt SA, Verhoef MJ, Arcury TA, Lewith GT, Steinsbekk A, Kristoffersen AE, Wahner-Roedler DL, Fønnebø V. Development of an international questionnaire to measure use of complementary and alternative medicine (I-CAM-Q). J Altern Complement Med. 2009 Apr;15(4):331-9. doi: 10.1089/acm.2008.0521.
