# Supplementary material for: Translation and cultural adaptation of the I-CAM-Q: the first Hungarian version for assessing complementary and alternative medicine use
Source: BMC Complement Med Ther. 2025 Dec 20;26:24. doi: 10.1186/s12906-025-05220-2 (PMC12837113; doi:10.1186/s12906-025-05220-2)
Supplement: Supplementary file 3 — Supplementary Material 3. [file 12906_2025_5220_MOESM3_ESM.docx]

***NAFKAM^[[1]](#footnote-1)^ Nemzetközi Komplementer és Alternatív Medicina Kérdőív (I-CAM-Q):***

***A KOMPLEMENTER ÉS ALTERNATÍV GYÓGYÁSZATI KUTATÁSOKHOZ AJÁNLOTT --***

***Önkitöltő változat***

1. ***Egészségügyi szolgáltatók felkeresése:*** Egészségügyi problémákkal különféle komplementer vagy konvencionális/hagyományos egészségügyi szolgáltatókhoz lehet fordulni.

| Járt-e már az elmúlt 12 hónapban az alábbi szolgáltatók valamelyikénél? | Igen  Nem  Hányszor kereste fel ezt a szolgáltatót az elmúlt 3 hónapban? | Kérjük, adja meg a szolgáltatónál tett *utolsó* látogatás *fő* okát  (Csak *az egyiket jelölje be.*). | | | | Mennyire volt hasznos számára, hogy felkereste ezt a szolgáltatót?  (Csak az egyiket jelölje be) |
| --- | --- | --- | --- | --- | --- | --- |
|  |  | Akut betegség/állapot esetén, amely egy hónapnál rövidebb ideig tartott | Krónikus (egy hónapnál hosszabb ideig tartó) egészségi állapot vagy tüneteinek kezelésére | A közérzet javítására | Egyéb  (Kérjük, adja meg az egyéb okot) |  |
|  |  |  |  |  |  | Nagyon  Némileg  Egyáltalán nem  Nem tudom |
| **Orvos** |  |  |  |  |  |  |
| **Csontkovács** |  |  |  |  |  |  |
| **Homeopata** |  |  |  |  |  |  |
| **Akupunktőr** |  |  |  |  |  |  |
| **Fitoterapeuta** |  |  |  |  |  |  |
| **Spirituális gyógyító** |  |  |  |  |  |  |
| **Egyéb szolgáltató:**  **____________** |  |  |  |  |  |  |
| **Egyéb (kérjük, adja meg):**  **____________** |  |  |  |  |  |  |
| **Egyéb (kérjük, adja meg):**  **____________** |  |  |  |  |  |  |

***2. (Orvosdoktori fokozattal rendelkező) orvosoktól kapott kiegészítő kezelések***

**Ha az elmúlt 12 hónapban nem járt orvosnál, kérjük, menjen tovább a 3. kérdésre.**

Egyes orvosok mind komplementer, mind konvencionális kezeléseket is nyújtanak.

| Az alábbi kiegészítő kezelések valamelyikével kezelte-e Önt orvos az elmúlt 12 hónapban? | Igen  Nem  Hányszor részesült ebben a kezelésben az elmúlt 3 hónapban? | Kérjük, adja meg a *fő* okot, amiért *legutóbb* ebben a kezelésben részesült  (Csak *az egyiket jelölje be.*) | | | | Mennyire volt hasznos az orvostól kapott kezelés?  (Csak az egyiket jelölje be.) |
| --- | --- | --- | --- | --- | --- | --- |
|  |  | Akut betegség/állapot esetén, amely egy hónapnál rövidebb ideig tartott | Krónikus (egy hónapnál hosszabb ideig tartó) egészségi állapot vagy tüneteinek kezelésére | A közérzet javítására | Egyéb  (Kérjük, adja meg az egyéb okot) |  |
|  |  |  |  |  |  | Nagyon  Némileg  Egyáltalán nem  Nem tudom |
| **Mozgás- és masszázsterápia** |  |  |  |  |  |  |
| **Homeopátia** |  |  |  |  |  |  |
| **Akupunktúra** |  |  |  |  |  |  |
| **Gyógynövény-alapú terápiák** |  |  |  |  |  |  |
| **Spirituális gyógyítás** |  |  |  |  |  |  |
| **Egyéb kezelés:**  **____________** |  |  |  |  |  |  |
| **Egyéb (kérjük, adja meg):**  **____________** |  |  |  |  |  |  |

***3. Gyógynövények és étrend-kiegészítők használata,*** beleértve a tablettákat, kapszulákat és folyékony gyógyszerformákat.

| Az alábbi kategóriákhoz soroljon fel legfeljebb három terméket, amelyet az elmúlt 12 hónapban használt. | Jelenleg használja ezt a terméket? | Kérjük, adja meg a *fő* okot, amiért *legutóbb* ebben a kezelésben részesült  (Csak *az egyiket jelölje be* ). | | | | Mennyire találta hasznosnak ezt a terméket?  (Csak az egyiket jelölje be.) |
| --- | --- | --- | --- | --- | --- | --- |
|  |  | Akut betegség/állapot esetén, amely egy hónapnál rövidebb ideig tartott | Krónikus (egy hónapnál hosszabb ideig tartó) egészségi állapot vagy tüneteinek kezelésére | A közérzet javítására | Egyéb  (Kérjük, adja meg az egyéb okot) |  |
|  |  |  |  |  |  | Nagyon  Némileg  Egyáltalán nem  Nem tudom |
|  | Igen  Nem |  |  |  |  |  |
| **Gyógynövények/Fitoterápia** | | | | | | |
| ________________ |  |  |  |  |  |  |
| ________________ |  |  |  |  |  |  |
| ________________ |  |  |  |  |  |  |
| **Vitaminok/Ásványi anyagok** | | | | | | |
| ________________ |  |  |  |  |  |  |
| ________________ |  |  |  |  |  |  |
| ________________ |  |  |  |  |  |  |
| **Homeopátiás szerek** | | | | | | |
| ________________ |  |  |  |  |  |  |
| ________________ |  |  |  |  |  |  |
| ________________ |  |  |  |  |  |  |
| **Egyéb étrend-kiegészítők** | | | | | | |
| ________________ |  |  |  |  |  |  |
| ________________ |  |  |  |  |  |  |
| ________________ |  |  |  |  |  |  |

***4. Önsegítő gyakorlatok***

| Használta-e az alábbi önsegítő gyakorlatok valamelyikét az elmúlt 12 hónapban? | Igen  Nem  Hányszor használta ezt a gyakorlatot az elmúlt 3 hónapban? | Kérjük, adja meg a *fő okot, amiért ezt az önsegítő gyakorlatot használta* (csak *egyet jelöljön be*). | | | | Mennyire találta hasznosnak ezt az önsegítő gyakorlatot?  (Csak az egyiket jelölje be) |
| --- | --- | --- | --- | --- | --- | --- |
|  |  | Akut betegség/állapot esetén, amely egy hónapnál rövidebb ideig tartott | Krónikus (egy hónapnál hosszabb ideig tartó tartó) egészségi állapot vagy tüneteinek kezelésére | A közérzet javítására | Egyéb  (Kérjük, adja meg az egyéb okot) |  |
|  |  |  |  |  |  | Nagyon  Némileg  Egyáltalán nem  Nem tudom |
| **Meditáció** |  |  |  |  |  |  |
| **Jóga** |  |  |  |  |  |  |
| **Csikung** |  |  |  |  |  |  |
| **Taj-csi** |  |  |  |  |  |  |
| **Relaxációs technikák** |  |  |  |  |  |  |
| **Vizualizáció** |  |  |  |  |  |  |
| **Részt vett hagyományos gyógyító szertartáson** |  |  |  |  |  |  |
| **Imádkozás a saját egészségért** |  |  |  |  |  |  |
| **Egyéb gyakorlat:**  **____________** |  |  |  |  |  |  |
| **Egyéb (kérjük, adja meg):**  **____________** |  |  |  |  |  |  |

**Forrás:** Quandt SA, Verhoef MJ, Arcury TA, Lewith GT, Steinsbekk A, Kristoffersen AE, Wahner-Roedler DL, Fønnebø V. Development of an international questionnaire to measure use of complementary and alternative medicine (I-CAM-Q). J Altern Complement Med. 2009 Apr;15(4):331-9. doi: 10.1089/acm.2008.0521.

1. NAFKAM: Norway's National Research Center in Complementary and Alternative Medicine (Norvég Nemzeti Komplementer és Alternatív Medicina Kutatóközpont) [↑](#footnote-ref-1)
